# Overweight and obesity among African immigrants in Oslo

**DOI:** 10.1186/1756-0500-6-119

**Published:** 2013-03-26

**Authors:** Abdi A Gele, Aneth J Mbalilaki

**Affiliations:** 1 Research Group for Inclusive Social Welfare Policies, The Department of Social Science, Oslo University College, Oslo, Norway; 2 Section for International Health, Department of General Practice and Community Medicine, University of Oslo, Oslo, Norway; 3 Institute of Sport and Biology, The Norwegian School of Sport Sciences, Oslo, Norway

**Keywords:** Overweight, Obesity, Immigrants’ health, Physical activity

## Abstract

**Background:**

Norway is experiencing an increase in overweight/obese adults, with immigrants from developing countries carrying a heavy burden. The aim of this study is to assess the prevalence of overweight and obesity among Somali immigrants in Oslo.

**Findings:**

A cross-sectional study involving 208 respondents aged 25 and over was conducted among Somali immigrants in Oslo, using a structured questionnaire. Prevalence of overweight/obesity varied by gender, with women having a significantly higher prevalence (66%) than men (28%). The mean BMI for females and males were 27.4 and 23.6, respectively. Similarly, 53% of women and 28% of men were abdominally obese. In a logistic regression analysis, both generalized and abdominal obesity were significantly associated with increasing duration of residence in Norway, and with being less physically active.

**Conclusion:**

Public health policymakers should facilitate an environment that enables Somali immigrants, particularly women, to lead healthy lifestyles. In this time of epidemiological transition, health education in the areas of physical exercise and healthy eating should be a major focus for working with new immigrants.

## Background

Obesity and overweight are reversible predisposing factors for the development of diabetes, hypertension and cardiovascular disease (CVD). An estimated 150 million people across Europe are obese, resulting in one million deaths and 12 million life years of ill health annually [1]. A rapid increase in the number of people developing obesity has been seen among the Norwegian population [2], with immigrants from developing countries being the most affected [3]. Factors associated with the host environment such as labor-saving technologies and the availability of high-calorie foods are frequently reported to be the causative factor of the obesity epidemic among immigrants [4]. Available epidemiological data on migration and health in Norway has increasingly focused on immigrants from Asia [3,5]. However, considerable heterogeneity in both obesity and overweight by region of birth has been documented [6]. Such classifications may be crucial in determining subgroups at higher risk for developing these conditions. A large Somali immigrant community exists in Oslo, the capital of Norway, yet little is known about prevalence and associated factors in overweight and obesity within this group.

Obesity and overweight are regarded as one of the five leading global risks for mortality in the world [7]. A prospective cohort study found that weight gain over time increases morbidity and mortality due to schematic heart disease (IHD). The same study attributed 27% of the incidence of IHD to weight gains ≥5 kg after the age of 18 years [8]. Apart from a relatively small number of people with specific metabolic disorders, the fundamental cause of overweight and obesity is positive energy balance resulting from the consumption of too much food in relation to the level of physical activity [9]. Post-migration obesity and overweight have been observed among immigrants in different settings [10]. Characteristics associated with living environments in a wealthy host country, e.g. a sedentary lifestyle and the availability of fatty foods, present immigrants with factors that can lead to overweight and obesity [11,12]. Changes in food habits after migration have been seen among immigrants in Oslo [13]. The fact that immigrants are less likely to receive counseling regarding good diet and exercise habits may place them in a more vulnerable position with respect to developing obesity over time. While this may be a common problem for Norway’s immigrants in general, differences in overweight and obesity among the various immigrant ethnic groups has been documented [3]. For example in Norway, overweight and obesity were higher among immigrants from Pakistan and Turkey compared to immigrants from Vietnam [3].

Somali immigrants comprise one of the largest asylum-seeking populations in both the Scandinavian countries and Norway. Physically, Somalis have slim bodies and a tall phenotype. Thus, a review article on obesity and overweight, in Eastern Mediterranean Region, reported the lowest mean BMI from Somalia, with mean BMI of 20.7 kg/m^2^ among men and 21.8 kg/m^2^ among women, while the highest mean BMI in the region was reported from Kuwait with mean BMI of 27 kg/m^2^ among men and 31 kg/m^2^ among women [14]. However, the lower BMI observed among Somalis in Somalia may have changed through immigration to Norway.

Despite progress over the past 20 years in reducing health inequality in Norway, ethnic, economic and other social disparities negatively affecting health still exist. While existing evidence supports the fact that Asian and African immigrants in Norway are chronically poor [15], individuals with a Somali ethnicity remain the most disadvantaged group in terms of employment compared to other first-generation immigrant groups, with Somali women being the most affected [16]. Obesity is strongly related to the socio-economic status of immigrants, with its prevalence increasing with increased levels of deprivation [17]. Thus, a recent qualitative study of 21 African immigrants in Oslo aged ≥50 years found that almost half of the Somali participants had diabetes or hypertension, or a combination of the two, thereby raising concerns about the magnitude of these conditions within this low-income immigrant community [18].

Perceptions towards body weight vary within communities and generations. Evidence reveals that Somalis do not consider overweight and obesity as a disease, but rather as a sign of success, wealth, good health and happiness [19,20]. A prior study among young Somali women in England shows that while young Somali women are conscious about having a healthy body size, they were constrained by older Somalis’ perception associating a large body size with self-esteem [21]. Moreover, Somalis have reported a cultural association with the concept of a relationship between fruits/vegetables and poverty, and between red meat and affluence [22]. Like those in other Western countries, the majority of Somali immigrants in Norway have moved from farming, pastoralism and labor-intensive lifestyles to urban settings that foster a more sedentary behavior [23]. Traditional Somali food rotates around rice, pasta, injera bread, meat, and tea with a lot of sugar; when this diet is combined with a sedentary lifestyle, it can quickly lead to overweight or obesity [24]. This pilot study investigates the prevalence of and associated factors in overweight/obesity among Somali immigrants in Oslo.

## Methods

Between June and August 2010, a cross-sectional study of Oslo’s ethnic Somali immigrant population was conducted. Oslo was selected as the study site because over 50% of Norway’s Somali immigrants reside there.

There are 600,000 immigrants in Norway, which constitutes 12.2% of the total population [25]. In Oslo, immigrants comprise 25% of the general population. While African immigrants constitute 12.3% of the immigrants in Norway, over 50% of them are Somalis, and an estimated 13,000 Somali immigrants currently reside in Oslo. Despite their large numbers in Oslo, no single study to date has investigated the prevalence and associated factors in overweight and obesity among this group. In response, the Diabetes Association of Norway funded this pilot survey to help gain insight into this important health issue. Because the sample frame for this group was unavailable, a non-random snowball sampling was employed to recruit the study sample. This sampling strategy has been used in previous studies of African immigrants when a random sampling strategy was unfeasible [26,27].

A pretested structured questionnaire (see Additional file 1) was consecutively administered to 208 Somali immigrants, with inclusion criteria being ≥25 years and giving consent for the participation of the study. The questionnaires were translated into Somali, the native language of both the researcher and the respondents. The questionnaire included socio-demographic information such as age, sex, marital status, education, occupation and years of stay in Norway. Lifestyle factors such as physical activity were also examined. Each person was asked to complete a short “in the last seven days” self-administered version of the International Physical Activity Questionnaire (IPAQ) [28]. The IPAQ was previously validated in Nigeria with communities that are comparable with Somalis [29]. Moreover, the questionnaires were translated into Somali and pretested prior to distribution to the sample. Responses were scored according to version 2.0 of the guidelines published at http://www.ipaq.ki.se. The diet of the participants was assessed by the response to the question: Do you consider your present diet to be mostly native, more fish and vegetable, or mixed? Studies on self-reported weight and height show an underestimation of weight and an overestimation of height [30]. Hence, in the present study, body mass index (BMI) and waist circumference (WC) were calculated from measurements taken by screening. Body mass index was calculated as weight (kg) divided by height squared (m^2^). There is no data to indicate which group, Asians, Caucasians or other Africans, is most appropriate to compare Somali data. However, a prior study, on overweight and obesity among Somali women in New Zealand, used the criteria for Europeans for Somali women as well [31]. Thus, obesity was defined as body mass index (BMI) ≥ 30 kg/m^2^ and overweight as BMI ≥ 25 kg/m^2^ <30 kg/m^2^. A waist circumference (WC) ≥88 cm for women and ≥102 cm for men was considered abdominally obese [32]. Prior to the interview, we ensured that each participant get clear idea about the study objectives, confidentiality of personally identifiable information and his/her right to withdraw from the study at any time. Afterwards, as large numbers of study participants were illiterate, verbal consent was obtained from all participants. The study was ethically cleared by the Norwegian Ethical Committee (REK).

### Data analysis

Data were analyzed using the statistics program SPSS, version 17.0. A statistical analysis was performed by calculating proportions of different people with different sociological and demographic characteristics. A chi-square was used for analyses of categorical variables, and a t-test or ANOVA for continuous variables. To determine the association between exposure and outcome variables, logistic regression analyses were conducted. First, univariate analyses were performed. The variables that have shown significant were then selected for multivariate logistic analyses with adjustment of age, education and marital status. The association was assessed using a 95% confidence interval (CI) and an adjusted odds ratio (aOR), and the level of significance was determined at a P-value <0.05.

## Findings

Our sample included 208 individuals: 93 (45%) males and 115 (55%) females. The difference in age and years of residence in Norway between males and females was not statistically significant. Overall, 104 (50%) were either overweight (35%) or obese (15%). Approximately 64% of overweight respondents were women. Similarly, of the 32 who were obese, 31 (97%) were women.

The mean BMI of the study sample was 25.7 (SE 0.28), with a significant difference in mean BMI observed between men (23.6) and women (27.4). The group differences in mean BMI are shown in Table 1. The mean WC was 95.29 cm (SE 0.9) for men and 92.55 cm (SE 1.19) for women. For this measure, 53% of the women had a WC ≥88 cm, while 28% of the men had a WC ≥ 102 cm. The difference in abdominal obesity between the males and females was statistically significant (P < .01).

**Table 1:** Crude mean BMI by gender, age, education, employment, years of stay in Norway, diet and physical activity

**Characteristics**	**N**	**%**	**Mean BMI (SE)**
**Gender**			
**Male**	93	44.7	23.6 (0.28)
**Female**	115	55.3	27.4 (0.29)**
**Age**			
**<33**	73	35.1	24.0 (0.40)
**33-40**	76	36.5	25.9 (0.42)
**>40**	59	28.4	27.6 (0.60)**
**Education**			
**University**	22	10.6	23.9 (0.70)
**Secondary**	84	40.4	25.1 (0.41)
**Primary/lower**	102	49.0	26.6 (0.43)**
**Employment**			
**Permanent emp.**	62	29.8	24.8 (0.53)
**Temporary emp.**	68	32.7	25.6 (0.49)
**Unemployed**	78	37.5	26.6 (0.45)*
**Diet**			
**More fruit/fish**	51	24.5	25.3 (0.56)
**Mixed**	62	29.8	25.6 (0.57)
**Native**	95	45.7	26 (0.40)
**Physical activity**			
**Vigorous**	7	3.4	24 (2.04)
**Moderate**	80	38.8	24.1 (0.40)
**Low**	119	57.8	27 (0.36)**
**Years in Norway**			
**0-4**	36	17.3	24.0 (0.44)
**5-9**	72	36.1	25.4 (0.41)
**10-14**	58	27.9	25.8 (0.59)
**>14**	42	27.7	27.7 (0.76)**
**Marital status**			
**Single**	30	14.4	23 (0.59)
**Married**	136	65.3	25 (0.33)
**Divorced/widowed**	42	20.2	27 (0.73)**

*Signifikant at <0.05.

**Signifikant at <0.002.

The adjusted logistic regression analysis shows that women were six times more likely than men to be overweight/obese (aOR 6.13, CI: 2.81-13.4). This difference was significant (p < .01). The mean BMI increased with the duration of residence in Norway. Respondents who have lived in Norway for more than 14 years had the highest mean BMI at 27.7 (overweight), and were seven times more likely to be overweight/obese than those who have lived in Norway for no more than four years (aOR 7.16, CI: 2.14-23.8). Similarly, those who reported not being physically active as measured by IPAQ were twice as likely to be overweight/obese as those who reported moderate or vigorous physical activity (aOR 2.19, CI: 1.06-4.49). Table 2 shows the association between BMI and the exposure variables.

**Table 2:** The association of BMI with exposure variables

**Variables**	**BMI <25**	**BMI** ≥ **25**	**Adjusted OR (95% CI)**
**Gender**			
**Male**	67 (72)	26 (28)	1.00
**Female**	39 (33.9)	76 (66)	6.13 (2.81-13.4)**
**Years in Norway**			
**0-4**	26 (72)	10 (27.8)	1.00
**5-9**	37 (51.4)	35 (48.6)	3.72 (1.30-10.61)*
**10-14**	30 (51.7)	28 (48.3)	2.56 (0.90-7.25)
**>14**	13 (31)	29 (69)	7.16 (2.14-23.8)**
**Physical activity**			
**Vigorous/moderate**	59 (67.8)	28 (32.2)	1.00
**No physical activity**	45 (37.8)	74 (62.2)	2.19 (1.06-4.49)*
**Diet**			
**More fish and vegetable**	29 (56.9)	22 (43.1)	1.00
**Mixed diet**	33 (53.2)	29 (46.8)	0.81 (0.32-2.03)
**Native diet**	44 (46.3)	51 (53.7)	0.72-3.84)

*Signifikant at <0.05.

**Signifikant at <0.002.

- Adjusted with age, education and marital status.

Regarding waist circumference, women were three times more likely than men to have a WC in the range of abdominal obesity (≥88 cm and ≥102 cm, respectively) (aOR 3.08, CI: 1.55-6.09), and respondents who were not physically active were twice as likely as the other groups to be abdominally obese (aOR 2.35, CI: 1.16-4.76). Likewise, the odds of being abdominally obese were about six times higher for those who had been in Norway for over 14 years as compared to their corresponding group (aOR 5.71, CI: 1.81-18.04). Table 3 shows the association between WC and the exposure variables.

**Table 3:** The association of WC with exposure variables

**Variables**	** Abdominal obesity (male ≥ 102 & female ≥ 88)**		**Adjusted OR (95% CI)**
	Obese N(%)	Not obese N(%)	
**Gender**			
**Male**	26 (28)	67 (72)	1.00
**Female**	61 (53)	54 (47)	3.08 (1.55-6.09)**
**Years in Norway**			
**0-4**	9 (25)	27 (75)	1.00
**5-9**	27(37.5)	45 (62.5)	2.33 (0.82-6.62)
**10-14**	23 (39.7)	35 (60.3)	2.07 (0.72-5.95)
**>14**	28 (66.7)	14 (33.3)	5.71 (1.81-18.04)**
**Physical activity**			
**Vigorous/moderate**	22 (25.3)	65 (74.7)	1.00
**No physical activity**	65 (54.6)	54 (45.4)	2.35 (1.16-4.76)*

*Signifikant at <0.05.

**Signifikant at <0.002.

- Adjusted with age and education.

## Discussion

The purpose of this study was to assess overweight/obesity among Somali immigrants in Oslo. The study demonstrates a significant difference between men and women in terms of BMI and WC. A mean BMI of 27.4 was found for the female respondents, which is higher than the mean BMI reported for Norwegian- (24), Sri Lankan- (26.7), Iranian-(26.2) and Vietnamese women (23.3) living in Norway. However, it is lower than the BMI reported for Turkish- (30.1) and Pakistani women (29.1) in Norway [33]. In this study, 66% of the women had a BMI indicating overweight or obesity (≥25 kg/m2), and 53% had a WC ≥88 cm. This is in line with a prior study in New Zealand which found that 71% of Somali women had a BMI ≥25 kg/m2 [31]. As such, this finding is not unexpected due to the fact that a high prevalence of generalized and abdominal obesity was found among Somali immigrants in different Western countries [19,22,31].

In the present study, participants who reported not being physically active were more than twice as likely than those who reported engaging in moderate or vigorous activity to have a BMI in the range of overweight or obese (≥25) (aOR 2.19, CI: 1.06-4.49), and were also twice as apt to be abdominally obese (aOR 2.35, CI: 1.16-4.76). Prior research has indicated that Somali immigrant women are concerned about the weight they have gained as a result of a lifestyle that is much more sedentary than the one they had in Africa [34]. In Oslo, Somali immigrants may receive limited or no counseling about physical exercise and diet. It is also noteworthy that access to physical activities that are familiar to or appropriate for Somali women is very limited. While being Muslim is not a barrier to women attending activities, the lack of appropriate facilities and information can constitute a barrier [35]. This study suggests that resources for nutrition and physical activity counseling should be committed to helping Norway’s African immigrants, particularly Somali women in Oslo.

The study also reveals that newly arrived (≤4 years) immigrants have a significantly lower mean BMI than those who have lived in Norway for more than 14 years (24 vs. 27.7). This finding is in line with other studies showing that overweight and obesity among immigrants increase with the length of time they live in the host country [14,36,37]. Nonetheless, it is not consistent with an earlier study of Asian immigrants in Oslo which showed that years of staying in Norway have had no impact on BMI changes [33]. This discrepancy may be a result of socio-cultural differences in immigrants’ countries of origin. For instance, over 80% of Somali people are pastoralists who rear livestock [38], so Somali immigrants were likely used to a labor-intensive lifestyle in their home country. It is therefore not surprising to see their BMI change the longer they live in the host country, where they have easy access to foods high in saturated fat, limited access to physical exercise aligned with their needs, and most importantly, a lack of proper health information to help them get adequate physical exercise. Problems underlying the high prevalence of overweight and obesity among Somali immigrants such as physical inactivity may not be an individual responsibility, but a result of interacting factors such as poverty, differences in culture and lifestyle, as well as poor delivery and uptake of health information. Providing appropriate health information on physical activity and diet in the early phase of an immigrant’s arrival could help arrest the gradual development of obesity in immigrant populations. Such counseling is likely to be more effective if directed at women since they are more likely than men to experience overweight/obesity with an increasing duration of residence in the host country.

Despite stringent efforts to make the sample as representative as possible, our snowball sampling strategy may have resulted in over-representation of those with more concerned on their body shape and size. The overall prevalence of overweight and obesity may therefore be underestimated. The relatively small sample size meant that the variables examined within the study were restricted to only few categories perhaps giving cruder indicators than desired. It is also possible that the study did not have the power to detect smaller, but possibly important, associations. However, given the difficulties of studying the Somali population in Norway, our data do provide much needed information on the social characteristics of a little studied group. Moreover, physical activity and diet, in addition to demographic variables such as education and years of stay in Norway, were assessed through self-reporting, thus implying the possibility of a recall bias. To minimize this potential bias, we used qualitative probing questions for the validation of respondents’ answers. Moreover, we used a non-probability sampling method; hence, the rates of overweight and obesity that were observed among the study sample may be different from the real situation in the general population.

## Conclusion

In this study, 66% of the female respondents had a BMI in the range of overweight and obese, and 53% were abdominally obese. This high prevalence of overweight\obesity among Somali women was associated with poor physical activity and increased duration of residence in Norway. Thus, public health policymakers should facilitate an environment that enables Somali immigrants, particularly women, to lead healthy lifestyles. Motivation for physical activity in the early phase of an immigrant’s arrival could help arrest the development of obesity among immigrants over the years. Moreover, the initiation of culturally sensitive physical activity centers might also have a substantial ameliorating impact on these adverse health conditions. In the meantime, a study on the barriers to and facilitators of physical activity among Somali women is required.

## Competing interests

The authors declare that they have no competing interests.

## Authors’ contributions

AG designed the protocol, carried out the field work, performed the data analysis, and drafted the manuscript. AJ participated in the design of the protocol, data collection, analysis, and drafting the manuscript. All authors read and approved the final manuscript.

## Supplementary Material

Additional file 1 Questionnaire for participants. Click here for file
