# Supplementary material for: Overweight and obesity among African immigrants in Oslo
Source: BMC Res Notes. 2013 Mar 26;6:119. doi: 10.1186/1756-0500-6-119 (PMC3617999; doi:10.1186/1756-0500-6-119)
Supplement: Additional file 1 — Questionnaire for participants. [file 1756-0500-6-119-S1.doc]

**Questionnaire for participants**

This questionnaire will be used as a tool for the research entitled **Risk factors for type 2 diabetes among Somali immigrants in Oslo**. The study is a pilot study that determines the prevalence of risk factors for type 2 diabetes among Somali immigrants in Norway. Your honest answers will help us improve the diabetes among African immigrants in Norway and other immigrant groups in general. We request your truthful answers, as it is not bad if your answer is either “I do not know or I don’t remember”.

Abdi A Gele and Julia Mbalilaki

Participant number................................ Date of interview...............................

Name of the interviewer......................................

**1. Personal and socio demographic information:**

1.1 **Age**.............years

1.2 **Sex**:

Male ……………………………………….□

Female ………………………………………□

1.3 **Education**

Unable to read and write ……………………□

Primary and lower …… ………………….□

Secondary …….. …………………………..□

College`\university………….……………….□

1.4 **Occupation**

Permanent job …………………………….. □

Temporary job …………………………... .□

Pension ………………………………….□

Unemployed ………………………………□

1.5 **Marital status**

Single ……………………………………..□

Married …………………………………… □

Divorced ………………………………….. □

Widowed………………………………….. □

1.6 **Years in Norway**

0-4 ……………………………………………□

5-9 ……………………………………………□

10-14 …………………………………………. □

>14 …………………………………………..□

1.7.  **Self rated health**

**How do you consider about your current health situation?**

Good ………………………………………… □

Fair ………………………………………….□

Poor ………………………………………….□

1.9*.* **Do you consider your diet mostly?**

Native ……… …………………………………□

More fish and vegetable……………………… ..□

Mixed …………………………………………..□

2. 0. **Smoking**

Yes…………………………………………..

No……………………………………………..

3. **Anthropometrical measures**

3.0  **WC**

Waist circumference _____ **cm**

Hip circumference _____**cm**

3.1. **BMI**

Weight………………….. ,……………………□

Length ………………………………………….□

4. **Physical activity (IPAQ)**

Think about all the **vigorous** activities that you did in the **last 7 days**. **Vigorous** physical activities refer to activities that take hard physical effort and make you breathe much harder than normal. Think *only* about those physical activities that you did for at least 10 minutes at a time.

1. During the **last 7 days**, on how many days did you do **vigorous** physical activities like heavy lifting, digging, aerobics, or fast bicycling?

_____ **days per week**

No vigorous physical activities ***Skip to question 3***

1. How much time did you usually spend doing **vigorous** physical activities on one of those days?

_____ **hours per day**

_____ **minutes per day**

□ **None**

Think about all the **moderate** activities that you did in the **last 7 days**. **Moderate** activities refer to activities that take moderate physical effort and make you breathe somewhat harder than normal. Think only about those physical activities that you did for at least 10 minutes at a time.

1. Again, think *only* about those physical activities that you did for at least 10 minutes at a time. During the **last 7 days**, on how many days did you do **moderate** physical activities like carrying light loads, bicycling at a regular pace, or doubles tennis? Do not include walking.

_____ **days per week**

No moderate physical activities ***Skip to question 5***

1. How much time did you usually spend doing **moderate** physical activities on one of those days?

_____ **hours per day**

_____ **minutes per day**

**None**

Think about the time you spent **walking** in the **last 7 days**. This includes at work and at home, walking to travel from place to place, and any other walking that you might do solely for recreation, sport, exercise, or leisure.

5. During the **last 7 days**, on how many days did you **walk** for at least 10 minutes at a time?

_____ **days per week**

No walking ***Skip to question 7***

1. How much time did you usually spend **walking** on one of those days?

_____ **hours per day**

_____ **minutes per day**

**None**

The last question is about the time you spent **sitting** on weekdays during the **last 7 days**. Include time spent at work, at home, while doing course work and during leisure time. This may include time spent sitting at a desk, visiting friends, reading, or sitting or lying down to watch television.

1. During the **last 7 days**, how much time did you spend **sitting** on a **week day**?

_____ **hours per day**

_____ **minutes per day**

□ **None**
